# Prolonged mHealth-Based Arrhythmia Monitoring in Patients With Hypertrophic Cardiomyopathy (HCM-PATCH): Protocol for a Single-Center Cohort Study

**DOI:** 10.2196/52035

**Published:** 2023-12-29

**Authors:** Sophia Schulze Lammers, Thorsten Lawrenz, Dennis Lawin, Annika Hoyer, Christoph Stellbrink, Urs-Vito Albrecht

**Affiliations:** 1 Department of Cardiology and Intensive Care Medicine University Hospital Ostwestfalen-Lippe of Bielefeld University Campus Klinikum Bielefeld Bielefeld Germany; 2 Department of Digital Medicine Medical Faculty Ostwestfalen-Lippe Bielefeld University Bielefeld Germany; 3 Biostatistics and Medical Biometry Medical Faculty Ostwestfalen-Lippe Bielefeld University Bielefeld Germany

**Keywords:** hypertrophic cardiomyopathy, nonsustained ventricular arrhythmia, sudden cardiac death, implantable cardioverter-defibrillator, long-term ECG, digital medicine, long-term electrocardiography

## Abstract

**Background:**

Patients with hypertrophic cardiomyopathy (HCM) are at increased risk of sudden cardiac death (SCD) due to ventricular arrhythmias and other arrhythmias. Screening for arrhythmias is mandatory to assess the individual SCD risk, but long-term electrocardiography (ECG) is rarely performed in routine clinical practice. Intensified monitoring may increase the detection rate of ventricular arrhythmias and identify more patients with an increased SCD risk who are potential candidates for the primary prophylactic implantation of an implantable cardioverter-defibrillator. To date, reliable data on the clinical benefit of prolonged arrhythmia monitoring in patients with HCM are rare.

**Objective:**

This prospective study aims to measure the prevalence of ventricular arrhythmias in patients with HCM observed by mobile health (mHealth)–based continuous rhythm monitoring over 14 days compared to standard practice (a 24- and 48-h long-term ECG). The frequency of ventricular arrhythmias in this 14-day period is compared with the frequency in the first 24 or 48 hours for the same patient (intraindividual comparison).

**Methods:**

Following the sample size calculation, 34 patients with a low or intermediate risk for SCD, assessed by the HCM Risk–SCD calculator, will need to be recruited in this single-center cohort study between June 2023 and February 2024. All patients will receive an ECG patch that records their heart activity over 14 days. In addition, cardiac magnetic resonance imaging and genetic testing data will be integrated into risk stratification. All patients will be asked to complete questionnaires about their symptoms; previous therapy; family history; and, at the end of the study, their experience with the ECG patch-based monitoring.

**Results:**

The Hypertrophic Cardiomyopathy: Clinical Impact of a Prolonged mHealth-Based Arrhythmia Monitoring by Single-Channel ECG (HCM-PATCH) study investigates the prevalence of nonsustained ventricular tachycardia (ie, ≥3 consecutive ventricular beats at a rate of 120 beats per minute, lasting for <30 seconds) in low- to intermediate-risk patients with HCM (according to the HCM Risk–SCD calculator) with additional mHealth-based prolonged rhythm monitoring. The study was funded by third-party funding from the Department of Cardiology and Intensive Care Medicine, University Hospital Ostwestfalen-Lippe of Bielefeld University in June 2023 and approved by the institutional review board in May 2023. Data collection began in June 2023, and we plan to end the study in February 2024. Of the 34 patients, 26 have been recruited. Data analysis has not yet taken place. Publication of the results is planned for the fall of 2024.

**Conclusions:**

Prolonged mHealth-based rhythm monitoring could lead to differences in the prevalence of arrhythmias compared to 24- and 48-hour long-term ECGs. This may lead to improved identification of patients at high risk and trigger therapeutic interventions that may provide better protection from SCD or atrial fibrillation–related complications such as embolic stroke.

**Trial Registration:**

Deutsches Register Klinischer Studien DRKS00032144; https://tinyurl.com/498bkrx8

**International Registered Report Identifier (IRRID):**

DERR1-10.2196/52035

## Introduction

Cardiac arrhythmias, especially nonsustained ventricular tachycardia (nsVT) and atrial fibrillation (AF), are important comorbidities of hypertrophic cardiomyopathy (HCM) [1,2]. In particular, nsVT is a predictor of sudden cardiac death (SCD) [3,4]. A well-established risk assessment is the HCM Risk–SCD calculator, which queries, among other parameters, the occurrence of nsVT. It is used for recommendations regarding the primary prophylactic implantation of an implantable cardioverter-defibrillator (ICD) [4].

Therefore, adequate screening for nsVT in patients with HCM is highly relevant. It must be assumed that more intensive monitoring can increase the detection rate of cardiac arrhythmias. For example, in one study, a 14-day long-term Holter electrocardiography (ECG) detected nsVT in up to 75% of intermediate-risk patients with HCM. In the same group, a 48-hour long-term ECG revealed nsVT in only 34% of patients [5,6]. In addition, a trial of 100 patients with HCM in Spain showed a 62% rate of nsVT during 30 days of monitoring compared to 8% in the first 24 hours [7]. In another study, 24- to 48-hour long-term Holter ECGs showed at least one AF episode in 23% of patients with HCM [8]. It can be speculated that prolonged ECG monitoring may increase AF detection, leading to the earlier introduction of oral anticoagulants to prevent embolic complications. Patients with HCM with AF have an exceptionally high risk of embolic complications [1].

The feasibility of mobile health (mHealth)–based screening for arrhythmias has not yet been thoroughly investigated in patients with HCM. Holter long-term ECGs and implantable loop recorders are established in everyday clinical practice. Whereas prolonged Holter monitoring is associated with patient discomfort, implantable devices are expensive, have some limitations concerning specificity in arrhythmia detection [9], and carry a risk of surgical intervention for implantation and explantation. More recently, mHealth ECG recorders have become increasingly available, and thus, it should be investigated to what extent mHealth-based prolonged ECG monitoring is feasible and accepted by patients.

In addition to the HCM Risk–SCD calculator, there is increasing evidence that the presence of late gadolinium enhancement and its extent is associated with an increased SCD risk in patients with HCM [10,11]. Furthermore, genotyping is recommended for patients with HCM by the current European Society of Cardiology guidelines since 60% of patients have mutations in cardiac sarcomere protein genes, which have been associated with an increased SCD risk [1,12]. Therefore, we aim to correlate the occurrence and frequency of nsVT associated with late gadolinium enhancement on cardiac magnetic resonance imaging (cMRI) or evidence of a pathogenic mutation. The extent of late gadolinium enhancement on cMRI will be graded as mild (<5%), moderate (5%-15%), or severe (>15%) using a semiquantitative score based on the standard left ventricular 17-segment model.

Prolonged monitoring for cardiac arrhythmias may have relevant implications for clinical decisions, such as primary prophylactic ICD implantation for nsVT and anticoagulation or rhythm-control measures for AF. The extent to which noninvasive prolonged rhythm monitoring through an mHealth ECG patch impacts the detection of cardiac arrhythmias and clinical decisions has not been investigated.

## Methods

### Study Design

The Hypertrophic Cardiomyopathy: Clinical Impact of a Prolonged mHealth-Based Arrhythmia Monitoring by Single-Channel ECG (HCM-PATCH) study aims to investigate the association between the occurrence of cardiac arrhythmias with detected gene mutations and the extent of late gadolinium enhancement in cMRIs. This single-center prospective cohort study will be conducted at the Department of Cardiology and Intensive Care Medicine at the University Hospital Ostwestfalen-Lippe (OWL), Campus Klinikum Bielefeld in Germany. The trial is registered under clinical trial number DRKS00032144 (DRKS-ID).

### Recruitment

According to the sample size calculation (see Statistical Analysis section), recruiting 34 patients will be sufficient for the statistical analysis. Study participants will receive an echocardiography at rest and during exercise (bicycle ergometry) and a cMRI. In addition, their symptoms, family history, and premedication are collected, and their HCM Risk–SCD calculator score will be calculated. According to this score, patients are allocated to three risk groups: low risk (<4%), intermediate risk (4%-6%), and high risk (>6%) of SCD within 5 years. An ICD is generally recommended in the high-risk group, should be considered in the intermediate-risk group, and is not indicated for the low-risk group [1]. For this study, it is planned to recruit 17 patients with low and 17 with intermediate risk scores. All these patients will receive the 14-day ECG patch.

Patients with HCM between the ages of 18 and 75 years will be included if they have not yet undergone implantable loop recorder, pacemaker, or ICD implantation and if their estimated 5-year SCD risk according to the HCM Risk–SCD calculator score is <6%. A maximum age of 75 years was set as the upper limit, as SCD in patients with HCM mainly occurs at a young age [13]. Patients with a pre-existing cardiac implant (pacemaker, ICD, or implantable loop recorder) or an already accepted indication for it; Morbus Parkinson disease with relevant tremor; a transcoronary ablation of septal hypertrophy procedure in the last 4 weeks; inability to give informed consent; and current symptoms or a history of skin cancer, rash from skin adhesive, or dermatoses (contraindication to wearing the patch) are excluded.

A therapeutic procedure is established for the patient based on the information available to date. Afterward, an ECG patch is applied to the chest to continuously monitor the heart rhythm over 14 days. After completing the 14-day wearing period, patients should return the ECG patch. Any arrhythmias recorded will then be documented, and the therapeutic procedure will be re-evaluated with a recalculation of the HCM Risk–SCD calculator score.

**Figure 1:**
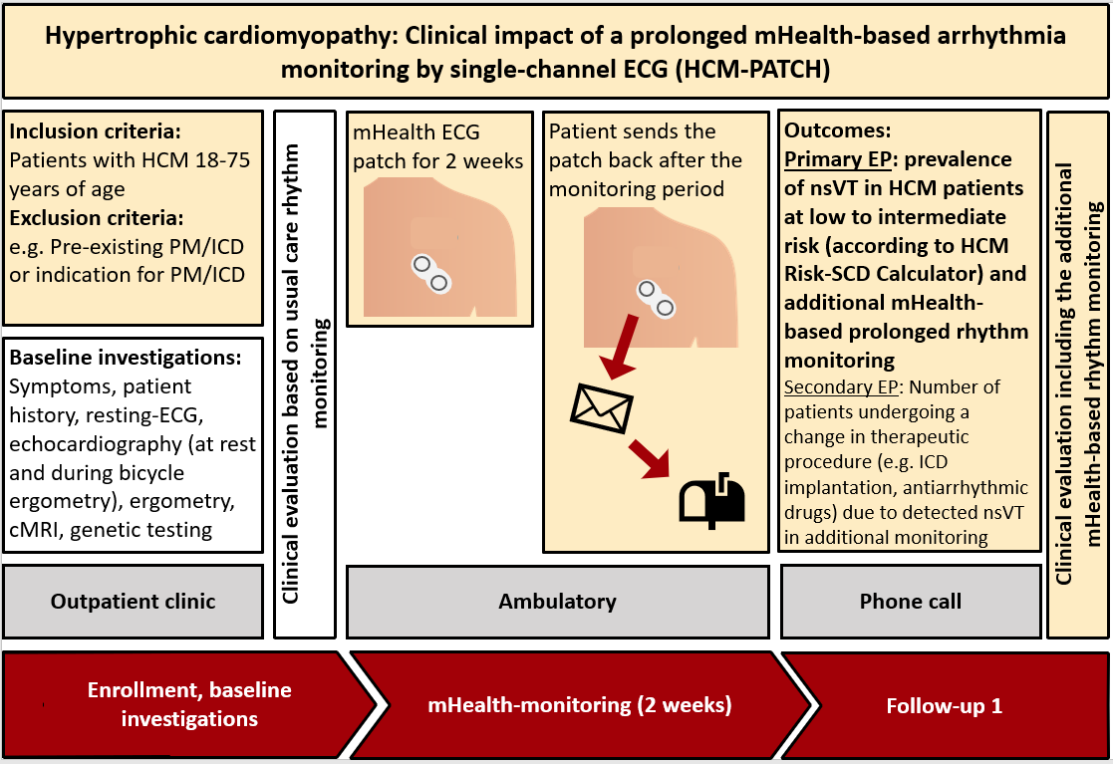
Overview of the study process. cMRI: cardiac magnetic resonance imaging; ECG: electrocardiography; EP: endpoint; HCM: hypertrophic obstructive cardiomyopathy; ICD: implantable cardioverter-defibrillator; mHealth: mobile health; nsVT: nonsustained ventricular tachycardia; PM: pacemaker; SCD: sudden cardiac death.

An overview of the study process can be seen in Figure 1.

### Questionnaires

At the beginning of study recruitment, various data such as the patient’s family history, previous syncope or rhythm abnormalities will be collected. In addition, secondary causes for left ventricular hypertrophy, such as arterial hypertension, will be queried. At the beginning and after monitoring completion, we will also ask patients if they have any questions, concerns, or requests they want to address with us. After completion of the 14-day monitoring period, the patient’s perception of the mHealth measure will be evaluated through a questionnaire (Multimedia Appendix 1). This questionnaire is based on the System Usability Scale by Brooke [14], a standardized questionnaire for evaluating utensils.

### Electrocardiogram Device

The ECG patch used is the AT-Patch from ATsens Co, Ltd (Seongnam-si, Republic of Korea), which is a single-channel ECG. The AT-Patch carries the CE mark as a registered medical device and is available for sale in the European Union. It can easily be attached to the chest and continuously records the heart rhythm for 14 days. During monitoring, patients can observe their ECG via an app (AT-Note) and may indicate symptoms (eg, dizziness or palpitations) via this app. Live tracking by the study physician is not possible. The ECG patch is light and compact and meets the grade of being showerproof (IP44/IP57). At the end of the monitoring period, the patient sends back the patch for rhythm evaluation. The detected arrhythmias are documented and adjudicated by two independent physicians.

**Figure 2:**
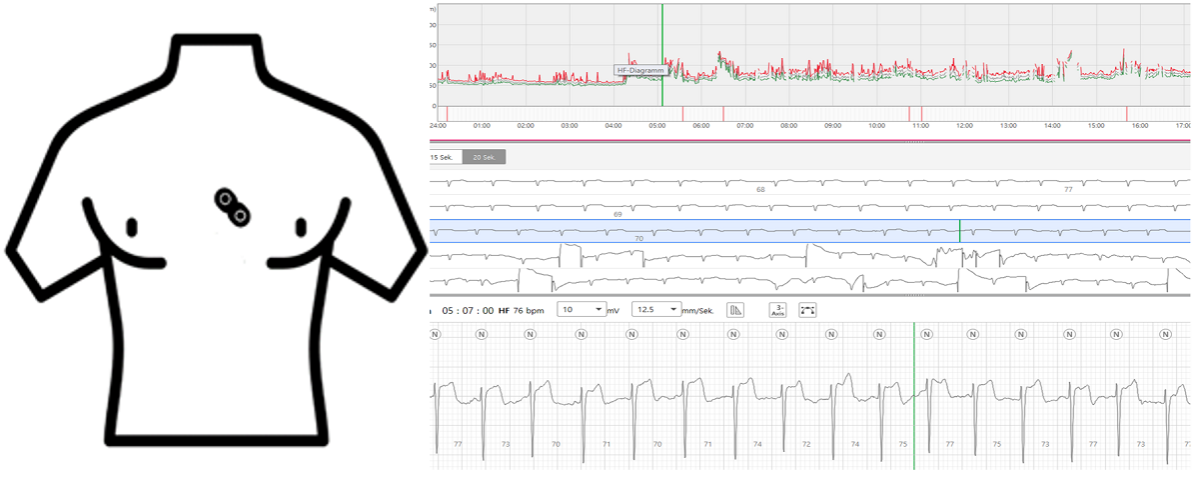
Single-channel electrocardiography system in situ and sample screen of the accompanying app.

Figure 2 shows a schematic representation of the ECG patch on the chest and an example rhythm analysis of the accompanying app.

### Outcomes

The rate of nsVT detection in the first 24 and 48 hours of monitoring versus the whole 14-day monitoring period will be compared. In addition, the prevalence of AF and other arrhythmias will be examined. Moreover, the results of the cMRI and, if applicable, the results of the genetic examination will be considered. The HCM Risk–SCD calculator score will be recalculated if new findings have occurred.

nsVT is defined as ≥3 consecutive ventricular beats at a rate of 120 beats per minute, lasting for <30 seconds [1]. AF is defined as atrial tachyarrhythmia lasting for >30 seconds [15]. The diagnosis of HCM is based on echocardiography; septum hypertrophy ≥15 mm in the absence of other causes of myocardial hypertrophy or septal or posterior wall thickness ratio >1.3 is required.

At the patients’ request, they will be informed about the rhythm-monitoring results and the resulting therapy changes after the ECG patch has been returned.

### Statistical Analysis

The sample size has been planned in collaboration with the Department of Biostatistics and Medical Biometry at the Medical School OWL, Bielefeld University. Patients should be divided into low- or intermediate-risk groups according to their HCM Risk–SCD calculator score, and both groups should be of equal size. Based on the prevalence of nsVT, an assumption was made for the group of low-risk patients based on the publications of Magnusson and Mörner [16] and Sakhi et al [17], where the prevalence after a 24- to 48-hour long-term ECG ranged from 7% to 42%. For case-finding purposes, the assumption was made that a similar increase (ie, a doubling) would be expected after 14 days in the intermediate-risk group. Therefore, a prevalence of 25% after 14 days is assumed. In the intermediate-risk group, a prevalence of 75% is expected [6]. Using a *χ*^2^ test, the total number of cases is 30, corresponding to 15 patients per group (α=0.05, power=0.8). In addition, a dropout probability of 10% was considered. This would result in 34 cases (17 per group).

After data collection, a statistical analysis will be conducted concerning the primary and secondary end points. Since this is a cohort study, all analyses will be adjusted for confounders, including, for example, age and sex. Potential confounders will be assessed from a clinical point of view before the statistical analysis is performed. A Poisson regression will be applied to analyze the primary end point (rate of nsVT detection). Furthermore, we will conduct a negative binomial regression as a sensitivity analysis. The same will be done for the secondary end points measured as the number of patients that experience specific events during the study period. Secondary end points regarding the prevalence of cardiac arrhythmias will be analyzed using logistic regression.

### Data Protection of Study Participants

The single-lead ECGs generated by the device are stored in the hospital network without external access. A patient identification list is kept in the Department of Cardiology, in which patient identification numbers are linked to the full patient names of the participants. This list allows later identification of participating patients. It will be kept confidential and archived for at least 10 years after the end of the study. The patient identification list will be stored separately from the documentation records on a computer using appropriate rights management and access monitoring. Subsequently, all patient-related data will be pseudonymized so that it is no longer possible for outsiders to identify individual patients. Passing on the pseudonymized data to external parties is expressly prohibited.

### Dropout

If the patient wishes to discontinue participation in the study during the 14 days of wearing the device, this is possible at any time and without any disadvantages for the patient. In addition, according to patients’ preferences, they may not be informed about rhythm observations. The study physician should be informed about this and discontinuation. If a patient does not return the patch after the 14 days, this will be counted as a dropout.

In addition, a minimum duration of 288 hours has been established. If the ECG patch is worn for less than this time, exclusion from the study will occur. How long the patch has been worn can easily be determined based on the duration that ECG data is available.

### Ethical Considerations

Each patient is informed about the nature, relevance, objectives, potential risks, expected benefits, implications, and other aspects of the clinical trial through a discussion between the investigator and the patient. The patients receive written patient information. The investigator makes sure that the patient has understood the information. After the information session, each patient is given sufficient time and opportunity to clarify any unanswered questions and to decide on their participation.

The study will be conducted following the ethical principles originating in the Declaration of Helsinki and the recommendations of Good Clinical Practice and Good Scientific Practice. In addition, the study was approved by the ethics committee of the Ärztekammer Westfalen-Lippe (2023-262-f-S).

The devices were partially purchased by our department and partially sponsored by the company ATsens. None of the manufacturers were involved in the study design, and they will not participate in the data collection, statistical analysis, or manuscript writing. There are no other financial contributions. Participation in the study is free of charge for all study participants.

## Results

The primary study objective and primary end point is the prevalence of nsVT in patients with HCM at low to intermediate risk (according to the HCM Risk–SCD calculator) and additional mHealth-based prolonged rhythm monitoring. We will also explore the following secondary end points:

- Number of patients undergoing a change in therapeutic procedure (ICD, antiarrhythmic drugs, etc) due to detected nsVT in additional monitoring
- Number of patients with detection of AF (duration >30 sec) by prolonged monitoring
- Number of patients who experience a change in therapeutic procedure (anticoagulation, antiarrhythmic drugs, catheter ablation, etc) due to detected AF
- Prevalence of cardiac arrhythmias in patients with positive cMRI findings (>5% late gadolinium enhancement)
- Prevalence of cardiac arrhythmias in patients with positive genetic diagnosis (detection of HCM-typical gene mutations)

Patient recruitment began in June 2023 and is expected to be completed in February 2024. Data analysis has not yet started. An example table listing patient characteristics and outcomes is provided in Multimedia Appendix 2.

## Discussion

This prospective study aims to measure the prevalence of nsVT in 34 patients with HCM observed by mHealth-based continuous rhythm monitoring over 14 days compared to standard practice (a 24- and 48-h long-term ECG). Thereby, the frequency of ventricular arrhythmias in the 14 days is compared with the frequency in the first 24 or 48 hours for the same patient (intraindividual comparison). We will evaluate whether the prolonged rhythm monitoring detects nsVT and AF more often compared to conventional 48-hour monitoring and to what extent these findings can induce changes in therapeutic management. It may be speculated that, especially in patients with intermediate HCM Risk–SCD calculator scores, prolonged monitoring may identify patients at high risk, who may potentially benefit from a prophylactic ICD, with higher accuracy. If no rhythm abnormality is detected during the 14-day monitoring, this will not likely occur in everyday life and ICDs can be avoided due to a sufficiently low risk. In addition, a higher diagnostic yield for previously undetected AF may also improve decision-making regarding the need for early anticoagulant therapy.

We set an upper age limit of 75 years because it is known that especially young patients with HCM especially have a higher risk of SCD [13]. Nevertheless, a residual risk remains, so no upper limit for ICDs is defined in the European Society of Cardiology guidelines. Several years ago, the DANISH (The Danish Study to Assess the Efficacy of ICDs in Patients With Nonischemic Systolic Heart Failure on Mortality) study demonstrated no survival benefit from an ICD in patients older than 75 years in a sample of patients with systolic heart failure [18]. We have followed this age limit despite the different patient samples.

We hypothesize that prolonged rhythm monitoring will detect more arrhythmias than 24- or 48-hour monitoring. In a study with prolonged rhythm monitoring by Holter ECG over 14 days, 75% of patients with HCM had nsVT, compared with only 16.9% of patients in the first 24 hours [6]. However, it remains unclear if all these patients with nsVT should now receive an ICD. Previous studies have shown that patients who present with nsVT during 24-hour monitoring show it more frequently if monitoring is prolonged. Patients who showed nsVT later in the subsequent 14 days usually had only one episode [7]. Thus, it can be speculated that the detection of nsVT in a 24-hour monitoring period indicated more frequent spells of nsVT overall, with an associated higher SCD risk. Moreover, the rate and duration of nsVT vary, suggesting that longer and more rapid nsVT episodes indicate a higher SCD risk [6]. Prolonged monitoring may allow for the identification of patients with more frequent nsVT episodes, also taking into account the rate and duration of nsVT.

In addition, a correlation with late gadolinium enhancement load on cMRI will be investigated. If patients with high late gadolinium enhancement load show increased nsVT, these scar areas detected on cardiac MRIs could be identified as possibly pathogenic for the arrhythmias, and appropriate therapy (eg, ICD fitting) should be performed. This has so far not been investigated, so the results are needed.

In addition, the mHealth ECG patch method used here offers opportunities for other diseases as well. For example, patients with a stroke of unclear origin can be screened for AF over an extended period. It is also possible to capture intermittent subjectively perceived cardiac arrhythmias with the help of such an ECG patch, as this is often not possible with normal long-term ECGs.

In summary, prolonged mHealth-based rhythm monitoring will be used to investigate arrhythmia burden in patients with HCM. Increased detection of arrhythmias compared with 24-hour long-term ECG monitoring can be expected. It is essential to individually consider the rhythm monitoring results and optimize therapy accordingly. Findings of prolonged rhythm monitoring might be helpful in cases of borderline risk. A thorough discussion of the findings and possibly individual reassessment using the risk calculator could be an approach.

## Appendix

Multimedia Appendix 1 The questionnaire to be used in the study.

Multimedia Appendix 2 Characteristics of patients with hypertrophic cardiomyopathy eligible for participation in the Hypertrophic Cardiomyopathy: Clinical Impact of a Prolonged mHealth-Based Arrhythmia Monitoring by Single-Channel ECG (HCM-PATCH) cohort study.

## Abbreviations

AF: atrial fibrillation
cMRI: cardiac magnetic resonance imaging
DANISH: The Danish Study to Assess the Efficacy of ICDs in Patients With Nonischemic Systolic Heart Failure on Mortality
ECG: electrocardiography
HCM: hypertrophic cardiomyopathy
HCM-PATCH: Hypertrophic Cardiomyopathy: Clinical Impact of a Prolonged mHealth-Based Arrhythmia Monitoring by Single-Channel ECG
ICD: implantable cardioverter-defibrillator
mHealth: mobile health
nsVT: nonsustained ventricular tachycardia
OWL: Ostwestfalen-Lippe
SCD: sudden cardiac death

## Data Availability

The data sets generated or analyzed during this study are not publicly available due to patient data protection but are available from the corresponding author upon reasonable request.
