# Supplementary material for: Prolonged mHealth-Based Arrhythmia Monitoring in Patients With Hypertrophic Cardiomyopathy (HCM-PATCH): Protocol for a Single-Center Cohort Study
Source: JMIR Res Protoc. 2023 Dec 29;12:e52035. doi: 10.2196/52035 (PMC10787333; doi:10.2196/52035)
Supplement: Multimedia Appendix 1 [file resprot_v12i1e52035_app1.docx]

**Questionnaire for the patients**

**Please answer the following statements on a scale from 1 (=I do not agree with the statement at all) to 5 (=****I fully agree with the statement).**

**I do not agree I fully agree**

| 1 | 2 | 3 | 4 | 5 |
| --- | --- | --- | --- | --- |

1. The ECG patch has limited me

in my daily life.

| 1 | 2 | 3 | 4 | 5 |
| --- | --- | --- | --- | --- |

1. I would use the ECG patch again in the future.

| 1 | 2 | 3 | 4 | 5 |
| --- | --- | --- | --- | --- |

1. I feel safer because of the prolonged surveillance than if there had been none.

| 1 | 2 | 3 | 4 | 5 |
| --- | --- | --- | --- | --- |

1. I found the ECG patch easy to use.

| 1 | 2 | 3 | 4 | 5 |
| --- | --- | --- | --- | --- |

1. I found the ECG patch more comfortable than a conventional long-term ECG.

(if you have never received a long-term ECG, please leave unanswered)
