# Supplementary material for: Prolonged mHealth-Based Arrhythmia Monitoring in Patients With Hypertrophic Cardiomyopathy (HCM-PATCH): Protocol for a Single-Center Cohort Study
Source: JMIR Res Protoc. 2023 Dec 29;12:e52035. doi: 10.2196/52035 (PMC10787333; doi:10.2196/52035)
Supplement: Multimedia Appendix 2 [file resprot_v12i1e52035_app2.docx]

**Table S1.** Characteristics of patients with HCM eligible for participation in the cohort study HCM PATCH

|  | | Value (n= 34) |
| --- | --- | --- |
|  | |  |
| **Basic Data** |  |  |
|  | Women, n (%) |  |
|  | Men, n (%) |  |
|  | Age (years) (median (Q1-Q3)) |  |
|  | Left atrial size on echocardiography (mm) (mean (SD)) |  |
|  | Maximum left ventricular wall thickness on echocardiography (mm) (mean (SD)) |  |
|  | Maximum LVOT^a^ Gradient on echocardiography (mmHg) (mean (SD)) |  |
|  | ECG-follow up time (h) (median (Q1-Q3)) |  |
| **Pre-Monitoring Analysis** |  |  |
|  | HCM-Risk SCD Score: low, n (%) |  |
|  | HCM-Risk SCD Score: intermediate, n (%) |  |
|  | Previous unexplained syncope, n (%) |  |
|  | Family History of sudden cardiac death, n (%) |  |
|  | NYHA^b^ I/II/III/IV, n (%) |  |
|  | Angina pectoris, n (%) |  |
|  | Diabetes mellitus, n (%) |  |
|  | Coronary artery disease, n (%) |  |
|  | Dyslipidemia, n (%) |  |
|  | Smoker, n (%) |  |
|  | Cerebrovascular accident, n (%) |  |
|  | Arterial hypertension, n (%) |  |
|  | Known Atrial Fibrillation, n (%) |  |
|  | Known non-sustained ventricular tachycardia, n (%) |  |
|  | Completed long-term ECG^c^ (n (%)) |  |
|  | Genetic status: negative/MYH7/MYBPC3/no genetic testing, n (%) |  |
|  | Left ventricular aneurysm, n (%) |  |
|  | Late gadolinium enhancement in cMRI^d^: none/mild/moderate/severe, n (%) |  |
|  | Treated with medication for HCM^e^, n (%) |  |
|  | - Beta blocker, n (%) |  |
|  | - Calcium channel blocker, n (%) |  |
|  | - Other, n (%) |  |
|  | Previous alcohol septal ablation, n (%) |  |
|  | Previous myectomy, n (%) |  |
| **Post-Monitoring Analysis** |  |  |
|  | Monitoring completed and ECG^d^ patch returned, n (%) |  |
|  | Time monitored (h)^a^ (mean (SD)) |  |
|  | Arrhythmia detected, n (%) |  |
|  | - Atrial Fibrillation detected, n (%) |  |
|  | - nsVT^f^ detected, n (%) |  |
|  | - nsVT^f^ detected in first 24 h, n (%) |  |
|  | - nsVT^f^ detected in first 48h, n (%) |  |
|  | - nsVT^f^ detected in first 7 d, n (%) |  |
|  | - other arrhythmia detected, n (%) |  |
|  | HCM-Risk SCD score: low, n (%) |  |
|  | HCM-Risk SCD score: intermediate, n (%) |  |
|  | Patients who were assigned to a different risk group based on monitoring, n (%) |  |
|  | Genetic status: negative/MYH7/MYBPC3/no genetic testing, n (%) |  |
|  | Evaluation of the patch as good/neutral/bad, n (%) |  |

a = left ventricular outflow tract, b = New York Heart Association, c = electrocardiogram, d = cardiac magnetic resonance imaging, e = hypertrophic cardiomyopathy, f= non-sustained ventricular tachycardia
